# Insights into the Thermo-Mechanical Treatment of Brewers’ Spent Grain as a Potential Filler for Polymer Composites

**DOI:** 10.3390/polym13060879

**Published:** 2021-03-12

**Authors:** Aleksander Hejna, Mariusz Marć, Daria Kowalkowska-Zedler, Agnieszka Pladzyk, Mateusz Barczewski

**Affiliations:** 1 Department of Polymer Technology, Gdańsk University of Technology, Narutowicza 11/12, 80-233 Gdańsk, Poland; 2 Department of Analytical Chemistry, Gdańsk University of Technology, Narutowicza 11/12, 80-233 Gdańsk, Poland; marmarc@pg.edu.pl; 3 Department of Inorganic Chemistry, Gdańsk University of Technology, Narutowicza 11/12, 80-233 Gdańsk, Poland; daria.zedler@pg.edu.pl (D.K.-Z.); agnieszka.pladzyk@pg.edu.pl (A.P.); 4 Institute of Materials Technology, Poznan University of Technology, Piotrowo 3, 61-138 Poznań, Poland; mateusz.barczewski@put.poznan.pl

**Keywords:** Brewers’ spent grain, modification, extrusion, wood polymer composites, color properties

## Abstract

This paper investigated the impact of twin-screw extrusion parameters on the properties of brewers’ spent grain. The chemical structure, antioxidant activity, particle size, and color properties, as well as the emission of volatile organic compounds during extrusion, were investigated. The main compounds detected in the air during modifications were terpenes and terpenoids, such as α-pinene, camphene, 3-carene, limonene, or terpinene. They could be considered as a potential threat to human health and the environment. Changes in the chemical structure, antioxidant activity, and color of materials after modification indicated the Maillard reactions during extrusion, which resulted in the generation of melanoidins, especially at higher temperatures. This should be considered an exciting feature of this treatment method because modified brewers’ spent grain may improve the thermooxidative stability of polymer materials. Moreover, the impact of the brewers’ spent grain particle size on color and browning index used to determine the melanoidins content was investigated. The presented results show that proper adjustment of extrusion parameters enables the preparation of brewers’ spent grain with the desired appearance and chemical properties, which could maximize the efficiency of the modification process.

## 1. Introduction

Brewers’ spent grain (BSG) is the most abundant by-product of the brewing industry. It stands for around 85% of them, significantly more than spent hops and excess yeast. Considering the size of the brewing industry, it should be considered as an important by-product. According to The Brewers of Europe organization and their European Beer Trends Statistics Report, 2019 Edition [1], the size of European production exceeds 42 billion liters of beer annually. Production of one hectoliter of the most popular beer, light lager, requires 20 kg of malt. Considering the growing popularity of other beer styles worldwide, the actual average value could be even higher [2]. After mashing, when starch is extracted from malt, the brewers’ spent grain stands for ~31 wt% of the initial malt weight. Therefore, around 6.2 kg of BSG is generated for each hectoliter of beer, which gives over 2.5 million tonnes for the whole of Europe [3]. Currently, this by-product’s main application is low-value animal feed with relatively low market value [4]. Brewers’ spent grain is commonly considered lignocellulose material since different fiber types account for over 70 wt% of its composition [5]. It is also rich in proteins, which account for around 20 wt% of BSG composition. Brewers’ spent grain often contains noticeable amounts of phenolics, providing additional antioxidant and antimicrobial properties to this by-product [6]. Moreover, BSG generated during the production of darker beers may contain noticeable amounts of melanoidins, the products of Maillard reactions. They are precious for food products because they enhance their shelf-life due to antioxidant and antimicrobial properties [7].

Because of its composition, brewers’ spent grain is often investigated as a potential food ingredient or additive [8,9]. However, the majority of works have been related to animal feed rather than human. According to the literature data, BSG positively impacted animal nutrition [10]. Nevertheless, the benefits of BSG application in human food products were also noted [11].

On the other hand, the composition of BSG makes it an auspicious raw material for the manufacturing of polymer composites. Moreover, the relatively similar composition was noted for other by-products applied as fillers for wood-polymer composites (WPCs) and raw materials for plywood or particleboards, such as wood flour or other agricultural wastes [12,13,14]. The presence of proteins may provide additional properties to the composite materials because they may act as plasticizers or take part in the Maillard reactions resulting in the generation of melanoidins [15].

However, there are two factors currently limiting the application of BSG in the manufacturing of WPCs. The first one is related to the high humidity of BSG, reaching even up to 25 wt% [15,16]. This makes BSG a perishable by-product. Nowadays, without its particular applications and recipients, it is not beneficial for breweries to perform additional drying of BSG. Another issue related to the applications of this by-product in WPCs production is the particle size. Although malts are ground before brewing, they contain noticeable amounts of the husk, which is beneficial for the filtration process after mashing. Therefore, considering the composite production, additional size reduction is required. The solution to overcome these drawbacks of BSG was developed [17]. In this paper, we describe the grinding of already dried BSG in a co-rotating twin-screw extruder. Application of extrusion enables the conducting of the process in a continuous manner, which is very beneficial from the industrial point of view—higher throughput and more straightforward implementation of results on an industrial scale [18]. Recent works associated with the extrusion treatment of lignocellulose materials confirm its potential [19,20]. In the presented paper, except for the resulting materials’ properties, we focused on the safety of this process. We investigated the emission of volatile organic compounds (VOCs) during extrusion treatment of brewers’ spent grain, which is very important and a crucial aspect of occupational safety and health [21]. We believe that investigating the impact of processes on human health and the environment is an integral part of their implementation into industrial practice.

## 2. Materials and Methods

### 2.1. Materials

Brewers’ spent grain used in the presented study was obtained from Energetyka Złoczew sp. z o.o. (Lututów, Poland). It was waste from the production of light lager and consisted solely of barley malts. The supplier had already dried the obtained BSG.

### 2.2. Extrusion Grinding of Brewers’ Spent Grain (BSG)

**Figure 1:**
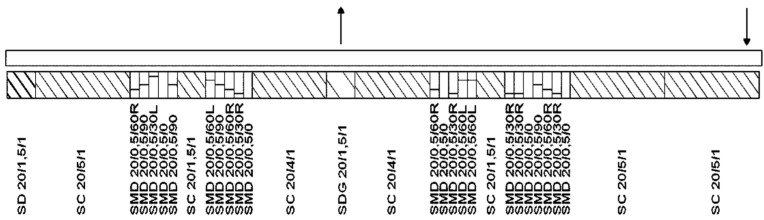
Screw configuration applied during extrusion grinding of brewers’ spent grain (BSG) (SMD kneading element, SC, SD and SDG types of conveying elements. The segments were named according to the Zamak Mercator specification).

**Figure 2:**
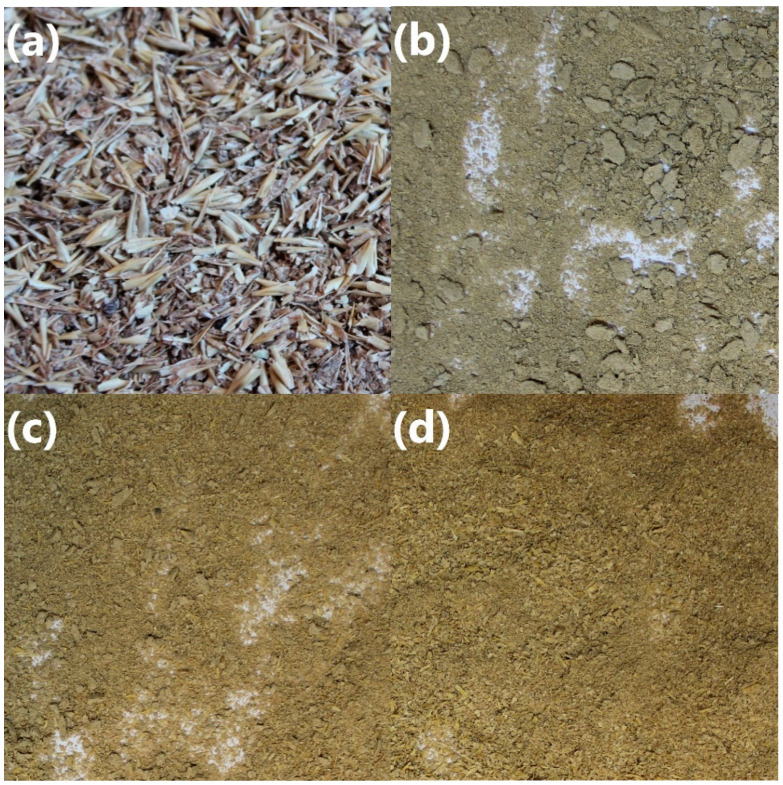
The appearance of (**a**) neat BSG, and extruded at (**b**) 60 °C, (**c**) 120 °C, and (**d**) 180 °C.

BSG was thermo-mechanically treated using an EHP 2 × 20 Sline co-rotating twin-screw extruder from Zamak Mercator (Skawina, Poland), according to our patent application [17] and our previous research work [15]. The extruder has 11 heating/cooling zones with a screw diameter of 20 mm and an L/d ratio of 40. Screw configuration is shown in Figure 1. BSG was dosed by a volumetric feeder with a constant throughput of 3 kg/h into the extruder barrel, while screw speed was fixed at 225 rpm for increasing throughput. Barrel temperature in all zones was set at 60, 120, or 180 °C. After grinding, samples of BSG were left in order to cool down to room temperature. Samples were coded as Neat BSG, 60, 120, and 180. Photographs of brewers’ spent grain before and after the extrusion at various temperatures are presented in Figure 2. The initial appearance of BSG points to its fibrous structure, associated with its origin. As mentioned above, BSG is generated during the mashing process, when the grain endosperm, consisting mostly of starch, is extracted [5]. Therefore, BSG is composed mostly of peel and pericarp, generally called the husk of grains [6]. After the extrusion grinding, the BSG still presents a fibrous structure. However, the average particle size is significantly smaller. The color changes are associated with the reduction of particle size and chemical reactions occurring during treatment. These factors are investigated and described in the presented paper.

### 2.3. Characterization Techniques

The screening studies on air quality near the workplace area were performed using a passive sampling technique. The samples of analytes present in the gaseous phase (workplace air) were collected by the Radiello^®^ diffusive passive samplers (Fondazione Salvatore Maugeri, Padova, Italy). The mentioned passive device is made of a diffusive membrane made of microporous polyethylene (PE) characterized by well-defined diffusion zone length, and the cylindrical container made of stainless steel mesh filled with graphitized charcoal, Carbograph 4. The type of diffusive membrane (color, porosity, diffusion zone) and applied sorption medium (strength and specific surface area) are designed for sampling of analytes classified as volatile organic compounds (VOCs). Detailed information about the application area and the operating parameters of this passive device might be found elsewhere [22,23,24]. The screening studies associated with the qualitative determination of VOCs present in the air near the investigated workplace were performed as follows: first, two Radiello^®^ diffusive passive devices were installed near the investigated workplace, at a distance of more than 1.5 m to avoid the phenomenon of competitiveness during the analytes sampling from gaseous phase; next, the exposure time of selected sampling devices in the investigated enclosed space was set up for 1.5 h, and the passive samplers were installed close to the investigated workplace and in the close range to the materials manufacturing area; during the whole sampling period the temperature was constantly monitored and oscillated between 21–22 °C; after the analytes sampling stage, stainless steel tubes were taken out from the PE cylindrical membrane and put into the glass tubes then sealed by a PE nut.

The identification of the VOCs collected on the applied sorption medium was performed employing the thermal desorption-gas chromatography-mass spectrometry (TD-GC-MS) system (Unity v.2, Markes International Ltd.; Pontyclun, UK), combined with gas chromatography (Agilent Technologies 6890) equipped with a mass spectrometer (5873 Network Mass Selective Detector, Agilent Technologies) working in the SCAN mode at the m/z range from 30 up to 450. In the beginning, the chemical compounds adsorbed on the sorption medium were thermally extracted at 280 °C for 15 min and transported directly to the microtrap (0 °C). After this, the chemical compounds were released from the microtrap sorption medium during the second thermal desorption stage by rapidly heating it to 300 °C and maintained by 5 min. Liberated from the microtrap, chemical compounds were transferred (helium flow rate—1.5 mL/min) directly to the GC column (Agilent 122-5563, J and W DB-5MS, 60 m × 0.25 mm × 1 μm). The GC oven works on the following temperature conditions: 50 °C for 1 min, then raised to 280 °C at a rate of 10 °C/min and then held at 280 °C for 10 min. The TD-GC and GC-MS transfer lines temperatures were 160 °C and 280 °C, respectively. Detailed information about the TD technique’s principles and characteristics was presented elsewhere [25,26]. Before each sampling period, the containers with Carbograph 4 were conditioned for 30 min at a temperature of 300 °C under an inert gas atmosphere. To assess signals that might be connected with other activities and processes in the investigated enclosed area and ensure the reliability of the results obtained, the “background” was also analyzed.

The particle size distribution of modified BSG samples was determined by sieve analysis. Samples were separated using LPzE-2e siever from MULTISERW-Morek Jan Morek (Brzeźnica, Poland) with sieves characterized by following openings: 2.0, 1.0, 0.8, 0.6, 0.4, 0.2, 0.1, 0.075, 0.053, and 0.025 mm. The total sieve time was set at 10 min, while the amplitude of pulsing was 2 Hz.

The chemical structure of brewers’ spent grain samples was determined using Fourier transform infrared spectroscopy (FTIR) analysis performed by a Nicolet Spectrometer iS50 from Thermo Scientific (Waltham, USA). The device had ATR attachment with the Specac Quest single reflection diamond attenuated total reflectance (ATR) accessory. Measurements were performed with 1 cm^−1^ resolution in the range from 4000 to 400 cm^−1^.

The color of ground organic powders was evaluated according to the Commission Internationale de l’Eclairage (CIE) through L*a*b* coordinates [27]. This system consists of three color components: L*—lightness (L* = 0 for black and L* = 100 for white), a*—the green(-)/red(+) component, b*—the blue(-)/yellow(+) component. Fifteen tests of each sample were undertaken and used for the determination of arithmetic mean values. The color was determined by optical spectroscopy using HunterLab Miniscan MS/S-4000S spectrophotometer from Hunter Associates Laboratory, Inc. (Virginia, USA), placed additionally in a specially designed light trap chamber. The following color parameters were determined based on the results obtained:

- total color difference parameter (ΔE*) calculated according to the following Equation (1) [28]:

ΔE* = [(ΔL*)^2^ + (Δa*)^2^ + (ΔL*)^2^]^0.5^ (1)

- chroma (C*_ab_) calculated according to the Equation (2):

C*_ab_ = [(a*)^2^ + (b*)^2^]^0.5^ (2)

- hue (h_ab_) calculated according to the Equation (3):

h_ab_ = tan^−1^ (b*/a*) (3)

Determined color parameters were also converted to the commonly used Adobe RGB color space defined by the three chromaticities of the red, green, and blue additive primaries [29].

Browning of the BSG resulting from thermo-mechanical treatment was assessed using two different methods. The first method was by calculating the browning index from the results of color analysis according to the following Equation (4) [30]:

BI = [(100 · (x − 0.31))/0.17] (4)

where: BI—browning index; x—calculated based on the Equation (5):

x = [(a* + 1.75 · L*)/(5.645 · L* + a* − 0.3012 · b*)](5)

The second method of browning index assessment was based on the approach presented by Supapvanich et al. [31]. The 0.1 g of the BSG sample was extracted by 20 mL of 65% (*v*/*v*) solution of ethanol. The solutions were left at room temperature overnight. The absorbance of extracts was measured at 405 nm using the Unicam SP300 spectrophotometer from Optima (Tokyo, Japan). Quartz cuvettes and 65% ethanol solution as a solvent were used.

The antioxidant activity (AA) of BSG samples was determined with a modified method described by Brand-Williams et al. [32] using synthetic DPPH radical (2,2-diphenyl-1-picrylhydrazyl). Samples of BSG were extracted with a 70% solution of ethanol for 20 h. Obtained extracts were centrifuged, and the activity of supernatants mixed with the proper amount of DPPH solution was determined by measurement of the absorbance at 517 nm. The percent DPPH scavenging effect was calculated by using the following Equation (6):

AA = I_%_ = [(A_0_ − A_s_)/A_0_ ] · 100% (6)

where: A_0_—absorbance for the blind test; A_S_—absorbance for an analyzed sample.

## 3. Results and Discussion

### 3.1. Volatile Organic Compounds Emissions during Extrusion of BSG

The emission of volatile organic compounds generated during various processes is an essential aspect of occupational safety and health. It is essential to investigate this issue during the development of industrial processes. Therefore, in the presented research work, we investigated the VOCs emissions during thermo-mechanical treatment of brewers’ spent grain in a twin-screw extruder using passive dosimetry. To exclude the emissions from other research activities performed on our laboratory hall and create the baseline emissions, we performed the background check. It resulted in the detection of multiple chemical compounds not associated with the extrusion of BSG but generated during storage and processing of natural and styrene-butadiene rubbers, polyethylene, polypropylene, polyurethanes, polyester resins, polystyrene, poly(vinyl chloride), and others—solvents and vulcanizing agents. These compounds were collected in Table 1, together with their boiling point and vapor pressure values, indicating that they can be considered a threat to human health. Although there is no strict definition of volatile organic compound and limit of vapor pressure, according to different organizations, VOC should be characterized by boiling point below 250 °C or vapor pressure exceeding 13 Pa [33,34,35]. Therefore, among the compounds listed in Table 1, only higher-molecular weight hydrocarbons are not classified as VOCs in a straightforward way.

**Table 1:** Compounds detected in the air during a background check of our laboratory hall.

Detected Compound	Chemical Formula	Chemical Structure	Boiling Point, °C	Vapor Pressure, Pa	Origin	Ref.
Hydrocarbons						
Hexane	C_6_H_14_	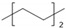	68	17,600	Polypropylene	[36,37]
Benzene	C_6_H_6_	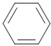	80	10,000	Styrene-butadiene rubber	[38]
Heptane	C_7_H_16_	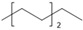	98	4600	Polyethylene, Polypropylene	[39]
Toluene	C_7_H_8_	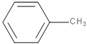	111	2800	Styrene-butadiene rubber, Solvents	[38]
*p*-Xylene	C_8_H_10_	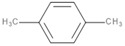	138	1200	Polyester resins, Styrene-butadiene rubber, Solvents	[38,40]
Ethylbenzene	C_8_H_10_	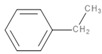	136	930	Polystyrene	[40]
Styrene	C_8_H_8_	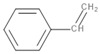	145	670	Polyester resins, Polystyrene, Styrene-butadiene rubber	[38,40]
Dodecane	C_12_H_26_	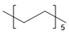	216	17	Natural rubber, Polyethylene, Polypropylene	[40,41]
Naphthalene	C_10_H_8_	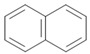	218	11	Poly(vinyl chloride)	[38]
Tridecane	C_13_H_28_	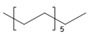	234	5	Polyethylene, Poly(vinyl chloride)	[38]
3-Methyltridecane	C_14_H_30_	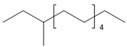	249	5	Polyester resins, Polyethylene, Polypropylene	[38,42]
Tetradecane	C_14_H_30_	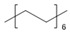	254	2	Polyester resins, Polyethylene, Polypropylene	[38,42]
Pentadecane	C_15_H_32_	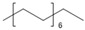	270	0.4	Polyester resins, Polyethylene	[38,42]
Hexadecane	C_16_H_34_	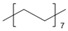	287	0.2	Polyethylene	[38,42]
Heptadecane	C_17_H_36_	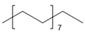	302	0.03	Polyester resins, Polyethylene	[38,42]
Chlorinated hydrocarbons						
Methylene chloride	CH_2_Cl_2_	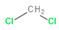	40	57,300	Polyurethanes, Solvents	[43]
Trichloromethane	CHCl_3_	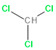	61	26,265	Solvents	[44]
Trichloroethylene	C_2_HCl_3_	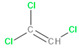	87	9200	Solvents	[44]
Tetrachloroethylene	C_2_Cl_4_	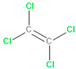	121	2470	Polyurethanes, Solvents	[43]
Ketones						
Acetone	C_3_H_6_O	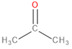	56	30,600	Solvents	[40]
Acetophenone	C_8_H_8_O	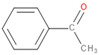	202	45	Polyethylene, Polystyrene	[42,45]
Aldehydes						
Benzaldehyde	C_7_H_6_O	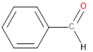	178	133	Polyester resins	[40]
Others						
Acetic acid	C_2_H_4_O_2_	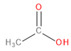	118	1520	Natural rubber, Polyethylene	[40,46]
Benzothiazole	C_7_H_5_NS	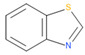	230	2	Vulcanizing agents	[47]
4-tert-Butylphenol	C_10_H_14_O	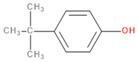	237	1.2	Poly(vinyl chloride)	[38]

The aim of the presented work was not only to determine VOCs emitted during the processing of BSG but also to assess the influence of the process on the surrounding environment. Therefore, for all detected compounds information is provided based on two commonly used international standards related to the safety of chemicals: the Globally Harmonized System of Classification and Labelling of Chemicals (GHS) and the NFPA 704: Standard System for the Identification of the Hazards of Materials for Emergency Response [48,49].

The GHS specifies the particular properties and their values, which are used to determine the threats posed by various chemicals. It includes physical, health, and environmental hazards towards humans and the environment. The information based on GHS is often included in safety datasheets of chemicals. For simplification, it describes chemicals with a combination of hazard statements and pictograms.

A slightly different approach is presented by the NFPA 704 system, which quantifies the hazards (from 0 to 4) related to the flammability, health threats, and chemicals’ instability. It can also provide information about the particular compound’s special hazards, associated, e.g., with strongly oxidative properties. Together, these four criteria create the “safety square” or “fire diamond”, which was developed to enable quick and efficient identification of the threats posed by particular chemicals.

In Table 2, there are listed VOCs detected during thermo-mechanical treatment of BSG in the extruder. The majority of detected compounds can be classified as terpenes or terpenoids. They are one of the largest natural product classes because they are generated during different biochemical transformations, mainly in plants [50]. They are composed of hydrocarbon backbone and often additional functional groups, such as carbonyls, carboxyls, or hydroxyls. They are often detected during the analysis of emissions from natural materials, such as wood or other grain products, e.g., malts [51,52]. Therefore, their presence among the VOCs emitted during thermo-mechanical processing of brewers’ spent grain is fully justified.

Although terpenes and terpenoids are natural compounds found in plants, they may threaten human health and the environment. These compounds may cause skin, eye, or respiratory irritation. Therefore, the GHS statements describing them include, e.g.,

- H304—May be fatal if swallowed and enters airways;
- H315—Causes skin irritation;
- H319—Causes serious eye irritation;
- H335—May cause respiratory irritation;
- H371—May cause damage to organs;
- H372—Causes damage to organs through prolonged or repeated exposure.

Considering the NFPA 704 standard, the health threats of detected compounds are classified mostly as 1 or 2, indicating that intense or chronic exposures may cause temporary incapacitation, possible residual injuries, or irritation with only minor effects. Only limonene was classified as 3, which is related to serious temporary or moderate residual injuries caused by short exposure. As there are no health hazards, rank 0 was given to 2,6-diisopropylnaphthalene, the only detected compound not included in terpenes or terpenoids. This chemical is applied industrially as a plant growth regulator by farmers [53]. Therefore, it was not detected in our previous work related to the cellulose and wood flour obtained mostly from the processing of conifers [54].

Nevertheless, despite the low risk to human health, the biodegradability of 2,6-diisopropylnaphthalene is very limited, so it can accumulate in plants and contaminate the natural environment [55]. It is described with H400 and H410 GHS statements pointing to the very toxic activity to aquatic life, even with long-lasting effects. Similar statements often describe α-pinene and camphene, rarely terpinene. Limonene and p-cymene are instead described with H411—Toxic to aquatic life with long-lasting effects. The differences between these statements are related to the lethal concentration (LC50) of chemicals, which for H400 and H410 is lower than 1 mg/L, while for H411 lower than 10 mg/L. At the same time, long-lasting effect indicated by H410 and H411 indicates the bioconcentration factor (BCF) higher than 500 [56].

The last factor described by the GHS and NFPA 704 standards are given in Table 2 as values of flash point which is the flammability of particular compounds. Terpenes and terpenoids are commonly known for their flammability because they are present in significant amounts in conifers, highly flammable wood species [57]. Detected compounds are classified according to GHS statement H226 indicating flammability in a liquid and gaseous state. NFPA 704 rated detected terpenes and terpenoids as 2 or 3, so they have to be moderately heated or exposed to relatively high ambient temperature to ignite. Technically, ratings 2 and 3 are related to the flash point in the range of 37.8–93.3 °C or 22.8–37.8 °C, respectively. Therefore, especially compounds rated as 3, so α-pinene, camphene, and terpinene, should be considered serious fire threats, and proper precautions have to be taken during the processing of lignocellulosic materials, including the monitoring of VOCs emissions.

### 3.2. Particle Size Distribution

**Figure 3:**
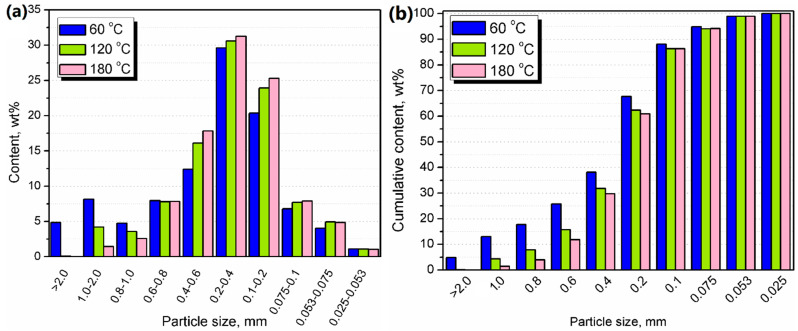
Graphs showing (**a**) content of particular fractions, and (**b**) cumulative particle size distribution of modified BSG samples.

In Figure 3, there is presented particle size distribution of the extruded brewers’ spent grain depending on the temperature of the process. It can be seen that the size of BSG particles depends on the applied temperature. Such an effect can be associated with the presence of moisture in the raw material. In the case of 60 °C, moisture evaporation was not so intensive as for higher temperatures. Therefore, agglomerates with dimensions exceeding 1 and even 2 mm were noted, which can be seen in Figure 2 showing the appearance of BSG. The agglomeration can be attributed to the intensification in hydrogen bonding between BSG particles [58]. No such effects were noted for higher temperatures, and the content of particles bigger than 1 mm was significantly lower, because of the moisture evaporation during processing, which limited the particle agglomeration [59]. Differences between 120 and 180 °C were smaller than between these temperatures and 60 °C since most moisture was removed. Moreover, the increase of the processing temperature noticeably enhanced the homogeneity of the material. Such an effect was probably associated with the lower moisture content and partial decomposition of the extractives and other low molecular weight components of BSG [60].

### 3.3. Color Properties

In Table 3, there are presented color parameters of the neat and extruded brewers’ spent grain samples and their antioxidant activity towards DPPH.

The appearance of particles, including their color, is directly influenced by the material’s physical structure. Considering color parameters, the one that is most significantly affected by the particle size is lightness [61]. The decrease in particle size is implicating the rise of the specific surface area. As a result, the reflection of light is enhanced, which increases the material’s lightness [62]. Therefore, unprocessed BSG was characterized by a significantly lower lightness value due to larger particle size (see Figure 2). It can be seen that the extrusion of BSG at 60 and 120 °C, which decreased the average particle size, caused a noticeable increase of lightness from 48.17 to 58.71. Nevertheless, such an effect was not noted when the temperature was increased to 180 °C leading to the lightness of 52.61. It was related to the non-enzymatic browning reactions occurring during the modification of BSG. These reactions include caramelization and Maillard reactions. Caramelization is a relatively complex process of simple sugars’ transformation. However, they are hardly present in BSG due to their removal during the mashing process. The Maillard reactions occur between amino acids and carbonyl groups of reducing sugars and result in a complex group of compounds, melanoidins [63]. These higher molecular weight oligomeric and polymeric substances are essential in food chemistry. Except for the color change, they are responsible for the enhanced storage stability of darker food products related to their antioxidant activity [64].

Contrary to the lightness, values of a* and b* parameters were not significantly affected by BSG extrusion temperature. These parameters are directly related to the color and hue of the material. All analyzed samples can be classified as brown, which was also expressed by similar hue values. Values of ΔE parameter, which include differences in L*, a*, and b* parameters, indicate large color variations resulting from the BSG treatment, according to PN-EN ISO 2813:2001 standard [28]. Nevertheless, it can be associated with a significantly larger particle size before extrusion grinding, which implicated noticeably lower material lightness (see Figure 2).

In Table 3 are also presented values of the browning index calculated from the color parameters. They indicate that samples extruded at 180 °C were characterized by the browning index’s highest value. According to the literature data [30], this factor can determine the extent of browning reactions during processing or drying of various products. Nevertheless, in this case, it was calculated from the color parameters of prepared samples which, as mentioned above, are sensitive to changes in particle size.

**Figure 4:**
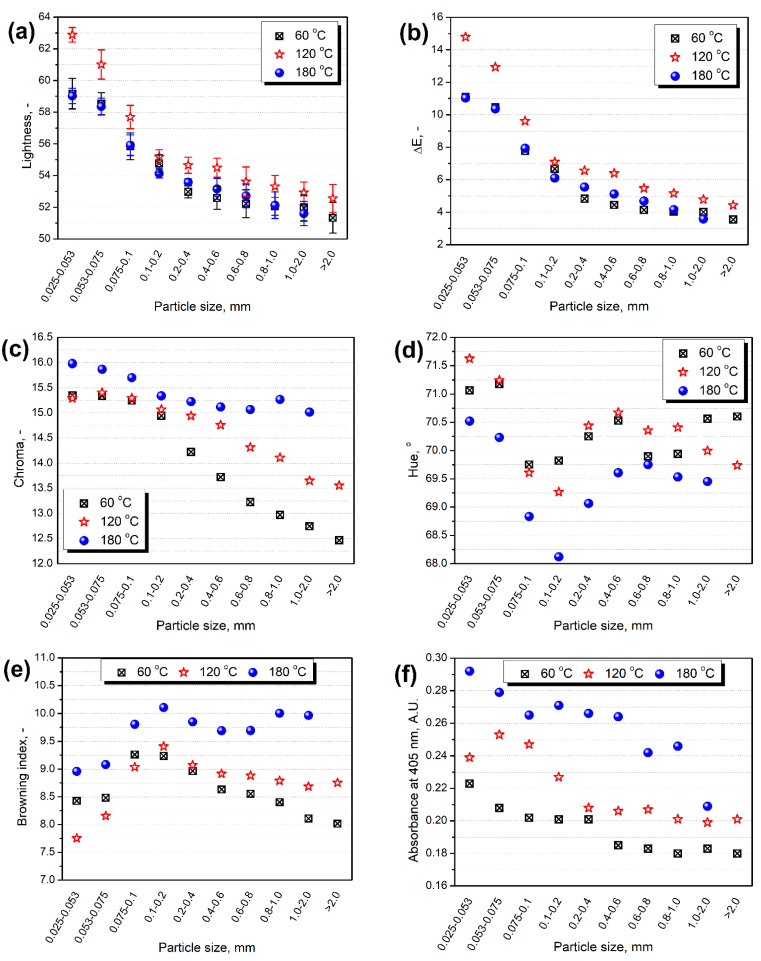
Plots of (**a**) lightness, (**b**) ΔE parameter, (**c**) chroma, (**d**) hue, (**e**) browning index, and (**f**) absorbance at 405 nm as a function of particle size of BSG.

To examine the changes in materials’ color without the impact of particle size, obtained samples were fractionated, and then their color parameters were determined. Figure 4a shows the lightness values of particular fractions of modified samples. It can be seen that the values of this parameter are significantly decreasing with the rise of the particle size, which is in line with the results presented by other researchers [65]. As a result, the values of ΔE parameter followed the same trend.

Also, other parameters, such as chroma and hue, are sensitive to the particle size. It can be seen that the chroma and hue were decreasing with the particle size of modified BSG. However, more direct dependence was noted for chroma. Similar findings for brown colors were described by Afoakwa et al. [66]. Regardless of the particle size range, chroma was increasing with the extrusion temperature. According to the findings of Morales and van Boekel [67] and Wu et al. [68], this indicates the increasing content of melanoidins, which are responsible for the browning of the material. The hue changes also confirmed this effect. Typical brown colors are characterized by its values in the range of 30–70° [66,69]. Figure 4e,f also confirmed the browning effect associated with the rise of the extrusion temperature. They point to the increase of browning index, independent of the selected method of its determination. Such effects are confirmed by other works, which indicate a significant rise in the high molecular weight melanoidin content with the reaction temperature and correlate it with the browning of material [70,71].

The browning of extruded BSG and suggested the rise of the melanoidin content resulted in the significant increase of the antioxidant activity measured by DPPH assay. For unmodified BSG, the value of antioxidant activity equaled 36%. Thermo-mechanical treatment at 60 and 120 °C caused its enhancement by 36% and 50%. However, by far the most significant effects were noted for the temperature of 180 °C, when antioxidant activity was almost doubled. Such an effect should be considered very beneficial for the potential applications of modified BSG as an antioxidant, e.g., in polymer technology [72]. A significant rise of the antioxidant activity could also be associated with brewers’ spent grain’s chemical composition. According to the data presented by Yilmaz and Toledo [73], BSG contains noticeable amounts of histidine, which results in high antioxidant activity of resulting melanoidins. Moreover, the analyzed by-product contains other antioxidants, such as ferulic acid or *p*-coumaric acid, which implicates relatively high activity even before modification [5].

### 3.4. Spectroscopic Analysis

**Figure 5:**
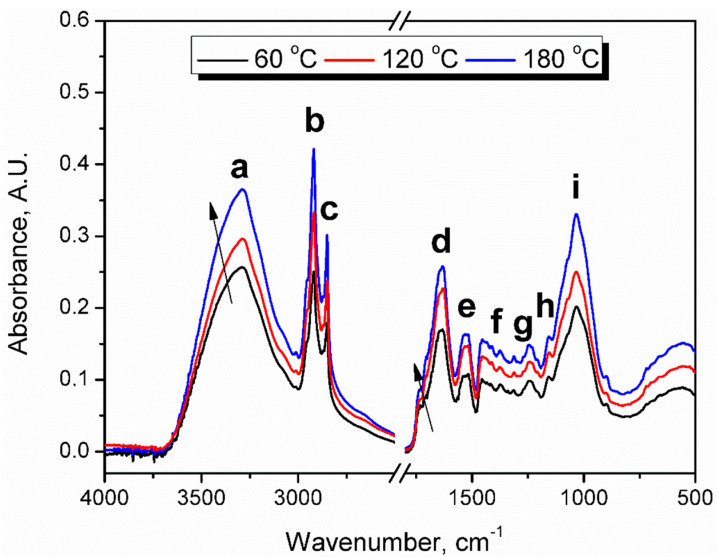
Fourier transform infrared (FTIR) spectra of extruded BSG.

Figure 5 presents FTIR spectra of extruded BSG samples. It can be seen that in qualitative terms, hardly any differences were noted between applied treatment temperatures. Generally, the same signals were noted for all samples, with only small shifts in their positions, within the range of 5 cm^−1^ (see Table 4). Observed spectra differed by the intensity of particular signals. However, they all showed an appearance typical for lignocellulose materials and were very similar to spectra obtained for melanoidins [74,75,76].

Strong signals (a) around 3291 cm^−1^ were attributed to O–H and N–H bonds’ stretching vibrations in hydroxyl, amine, and amide groups. Hydroxyl groups are commonly present in all types of lignocellulose materials. The presence of amines and amides is associated with the high content of proteins in brewers’ spent grain and their reactions with sugars generating melanoidins. The signals (a) were not very sharp because amide groups are often noted above 3300 cm^−1^ [77]. Moreover, broad signals indicate hydrogen bonding between O–H and N–H groups [78].

Peaks characteristic for the symmetric and asymmetric stretching vibrations of C–H bonds can be observed at 2850 and 2919 cm^−1^. The increase in their magnitude may result from the generation of higher-molecular weight melanoidins [76]. Another very strong signal was noted at 1633 cm^−1^, attributed to the stretching vibrations of C=O, C=N, and possibly C=C double bonds [79]. This signal’s intensity is clearly increasing with the rise of BSG processing temperature, which indicates the generation of amide groups in melanoidins during Maillard reactions [75]. Moreover, the observed rise of intensity could confirm the enhancement of the antioxidant activity of modified BSG by the rise of extrusion temperature. The positive correlation of this factor with the magnitude of the 1630 cm^−1^ peak was noted by Mot et al. [80], as well as Oracz and Zyzelewicz [76]. They ascribed this signal not only to the C=O and C=C vibrations in melanoidins but also in flavonoids. Mot et al. [80] even suggested that this signal could be used as a parameter for measuring antioxidant activity. Broadening of the peak at 1633 cm^−1^ was noted due to overlapping with a relatively weak peak at 1730–1740 cm^−1^ was noted, which was also associated with C=O stretching. However, its low magnitude was related to the fact that this moiety is not present in all types of melanoidins [75]. Absorption bands (e) in the range of 1517–1533 cm^−1^ were associated with the amide II vibrations, as well as C=C stretching vibrations of aromatics [81]. Multiple weak signals (f) observed at 1412–1456 cm^−1^ were related to the C–H bending vibrations in hydrocarbon chains [74]. Similar signals (g) characteristic for O–H bending interacting with C–N and C–O stretching were observed at 1242–1312 cm^−1^ [82]. Peaks (h and i) at 1032–1034 and 1155–1157 cm^−1^ were related to the stretching vibrations of C–N and especially C–O bonds.

## 4. Conclusions

The presented paper aimed to investigate the impact of the parameters of twin-screw extrusion grinding on the performance of brewers’ spent grain to evaluate its usefulness in different applications, mainly as a filler for polymer composites. Chemical structure, antioxidant activity, particle size, and color properties were investigated. Moreover, the emission of volatile organic compounds during extrusion was analyzed in qualitative terms. The main detected compounds were terpenes and terpenoids, which may threaten human health and the environment. These compounds cause skin, eye, or respiratory irritation, may be toxic to aquatic life and are often characterized by high flammability. Some of them show relatively low values of flash point, even below 40 °C. In further works, we would also include the quantitative assessment of VOCs emissions, which would enable a full evaluation of the potential fire threats. Nevertheless, even results presented in this paper indicate that serious precautions need to be taken during the modification of lignocellulosic fillers involving elevated temperatures or high shear forces.

The increase of extrusion temperature caused a drop in the particle size of modified BSG. Moreover, it eliminated the agglomeration of particles noticed at 60 °C, which was related to moisture content, not reduced during treatment. Considering their potential applications in the manufacturing of polymer composites, such an effect is very beneficial. On the other hand, in food processing, the granulation is often the desired result, which indicates that BSG modified by the investigated process could find application in various industry branches.

Changes in the color of brewers’ spent grain after modification, together with the analysis of chemical structure and assessment of antioxidant activity, indicate the generation of melanoidins during the extrusion treatment, especially at elevated temperatures. After extrusion at 180 °C, antioxidant activity was even doubled compared to the reference sample and 35% higher than after processing at 120 °C. Such an effect can be considered very beneficial for potential applications because melanoidins could enhance the thermooxidative stability of polymer materials, which would be evaluated in further works.

Moreover, the dependencies between the particle size of modified BSG and their color parameters, as well as the browning index used to determine the melanoidins content, were analyzed. Such analysis indicates that the resulting material could be fractionated and directed for different products or applications, which could maximize the efficiency of the modification process.

The presented paper confirmed that the twin-screw extrusion could be regarded as an effective method for grinding brewers’ spent grain to use it to manufacture polymer composites. Nevertheless, the results obtained indicate that extruded BSG may also be considered an exciting material for the food industry, mainly due to the relatively high antioxidant activity. The changes in the extrusion parameters can adjust the properties of the resulting material. Moreover, compared to our previous works, we have taken one step towards the industrial application of the BSG extrusion by the qualitative assessment of volatile organic compounds’ emissions during the process. Further works would also include the quantitative evaluation which, together with the exposure limits, would determine if the investigated process could be safely implemented in industrial practice. However, before the potential implementation, a couple more issues should be addressed:

- the use of energy and water during extrusion should be determined for different parameters of the process, which would enable economic calculations and life-cycle assessment analyses;
- depending on the final application of modified BSG, its impact on the properties of polymer composites or various food products should be evaluated, especially considering the antioxidant activity of melanoidins generated during treatment;
- the possibility of incorporating additional compounds improving the performance of modified BSG in various applications should be evaluated.

## Figures and Tables

**Table 2:** Volatile organic compounds detected during the extrusion treatment of BSG.

Compound	Formula	Chemical Structure	Vapor Pressure, Pa	Boiling Point, °C	Flash Point, °C	NFPA 704 Codes			GHS Pictograms
						*F*	*H*	*I*	
o-Cymene	C_10_H_14_	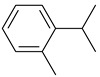	200	178	50	2	1	0	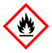
*m*-Cymene	C_10_H_14_	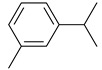	200	175	47	2	1	0	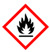
*p*-Cymene	C_10_H_14_	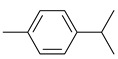	200	177	47	2	1	0	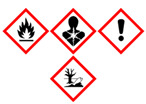
-Pinene	C_10_H_16_	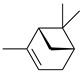	633	156	33	3	1	0	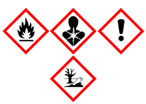
Camphene	C_10_H_16_	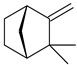	333	160	34	3	2	1	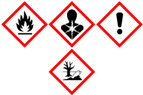
3-Carene	C_10_H_16_	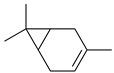	496	170	46	2	2	0	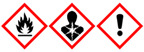
Terpinene	C_10_H_16_	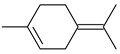	99	186	37	3	2	2	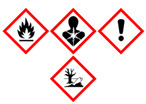
Limonene	C_10_H_16_	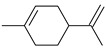	190	176	50	2	3	0	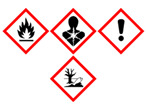
Camphor	C_10_H_16_O	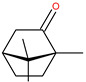	27	209	54	2	2	0	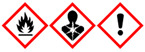
2,6-Diisopropylnaphthalene	C_16_H_26_	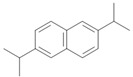	0.07	279	140	1	0	0	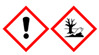

**Table 3:** Color parameters of the neat and extruded BSG.

Sample	Color Parameters											AA, %
	L*	a*	b*	ΔE*	R	G	B	Color	C_ab_*	h_ab_, °	BI	
BSG	48.17	5.11	13.04	-	124.8	110.3	93.8		14.0	68.6	10.22	36
60	54.08	4.96	13.75	5.96	139.9	124.7	106.5		14.6	70.2	9.05	49
120	58.71	4.72	13.80	10.57	151.7	136.5	117.6		14.6	71.1	8.06	54
180	52.61	5.55	14.66	4.74	137.1	120.7	101.6		15.7	69.2	10.27	71

**Table 4:** Detailed information about the signals observed in the FTIR spectra of analyzed materials.

Signal	Sample			Origin
	60	120	180	
	Wavenumber, cm^−1^			
a	3291	3290	3290	O–H, N–H stretching
b	2919	2919	2119	C–H asymmetric stretching
c	2851	2850	2850	C–H symmetric stretching
d	1633	1633	1632	C=O, C=N, C=C stretching
e	1517–1532	1517–1532	1518–1533	N–H bending, C=C, C–N stretching
f	1417–1456	1413–1455	1412–1454	C–H bending
g	1242–1312	1243–1312	1247–1312	O–H bending, C–N, C–O stretching
h	1157	1156	1155	C–N, C–O stretching
i	1032	1034	1034	C–N, C–O stretching

## Data Availability

Data is contained within the article. The data presented in this study are available in Insights into the thermo-mechanical treatment of brewers’ spent grain as a potential filler for polymer composites.
